# Endocrine manifestations of Madelung disease: A rare case report presenting with life-threatening alcohol-related hypoglycemic coma

**DOI:** 10.1097/MD.0000000000049569

**Published:** 2026-07-03

**Authors:** Yu Zeng, Song Huang, Jingyu Hu

**Affiliations:** a Department of Endocrinology, Longchang People’s Hospital, Longchang City, Sichuan Province, China; b Department of Critical Care Medicine, Longchang People’s Hospital, Longchang City, Sichuan Province, China.

**Keywords:** alcoholic hypoglycemia, benign symmetrical lipomatosis, coma, hypoglycemia, Madelung disease

## Abstract

**Rationale:**

Madelung disease (MD), also known as multiple symmetrical lipomatosis, is a rare benign lipid metabolism disorder characterized by the gradual development of symmetrical, non-encapsulated lipomatous hyperplasia, primarily affecting the subcutaneous adipose tissue of the neck, shoulders, trunk, and extremities. Although existing literature has thoroughly elucidated the typical histomorphology and clinical manifestations of this disease, cases presenting with life-threatening metabolic complications as the initial clinical presentation are extremely rare, which poses significant challenges for early clinical diagnosis and timely intervention.

**Patient concerns:**

A 62-year-old male presented with abrupt altered consciousness lasting more than 2 hours, with a long history of heavy daily alcohol intake, chronic alcoholic liver disease and gradually enlarged bilateral neck masses for 10 years.

**Diagnoses:**

1. hypoglycemic coma; 2. MD.

**Interventions:**

The patient was intravenously administered 40 mL of a 50% glucose solution in the emergency department. After admission, continuous blood glucose monitoring was performed and intravenous glucose infusion was continued. The patient also received standardized hepatoprotective and lipid-lowering treatment.

**Outcomes:**

Following prompt glucose supplementation, the patient regained full consciousness, achieved stable blood glucose control, and demonstrated improved liver function before hospital discharge. The patient declined surgical resection for MD, and no surgical intervention was performed for this admission.

**Lessons:**

This case describes an extremely rare clinical scenario where chronic excessive alcohol consumption serves as the primary cause of hypoglycemic coma, while hepatic steatosis from Madelung-related diffuse lipomatosis aggravates impaired hepatic gluconeogenesis. It further highlights the critical importance of including MD in the differential diagnosis for patients presenting with unexplained coma who also exhibit characteristic symmetrical neck lipomatous hyperplasia. Timely intravenous glucose supplementation can rapidly reverse severe hypoglycemia and save lives; clinicians should maintain a high index of suspicion for this combined disorder in chronic alcoholic patients.

## 1. Introduction

The German surgeon Otto Madelung first described Madelung disease (MD) in 1888. This rare condition, whose etiology remains unclear, is also termed benign symmetrical lipomatosis or Launois–Bensaude syndrome.^[1]^ Nevertheless, clinicians can reliably diagnose the condition by relying on 3 key pillars: a detailed medical history, a physical examination revealing its pathognomonic adipose deposits, and corroborative evidence from imaging studies.^[2]^ We present a case where hypoglycemic coma, predominantly induced by chronic alcohol intake with MD-induced liver injury as an aggravating factor, served as the initial manifestation of MD, highlighting a potentially life-threatening association. This report underscores the necessity of including MD in the differential diagnosis of unexplained coma, particularly in patients with suggestive physical signs and long-term heavy drinking history. Enhanced recognition of this acute metabolic manifestation is imperative to avoid diagnostic delays, guide appropriate intervention, and ultimately mitigate significant morbidity.

## 2. Case details

A 62-year-old male was admitted due to “sudden-onset impaired consciousness for over 2 hours.” Approximately 2 hours before arrival, he developed an abrupt alteration in mental status without identifiable triggers. The patient was unresponsive to verbal stimuli, with no accompanying limb convulsions, oral frothing, gaze fixation, or urinary/fecal incontinence. Emergency medical services were activated, and the patient was transported to the emergency department. Point-of-care blood glucose testing revealed severe hypoglycemia (1.2 mmol/L). His consciousness was gradually restored after intravenous administration of 50% glucose.

Past medical history included hypertension for 3 years, with a recorded peak blood pressure of 231/151 mm Hg (1 mm Hg ≈ 0.133 kPa), although the current antihypertensive regimen and control status were unknown. The patient also had a 10-year history of alcoholic liver disease. Social history indicated a 20-year smoking history and a 20-year history of daily alcohol consumption, primarily baijiu (Chinese spirits), averaging approximately 200 mL per day. The patient reported progressive anterior and posterior neck masses for 10 years, which had never been medically evaluated. He also had a prior episode of hypoglycemic coma, although the specific clinical details and management remain unclear.

**Figure 1.**
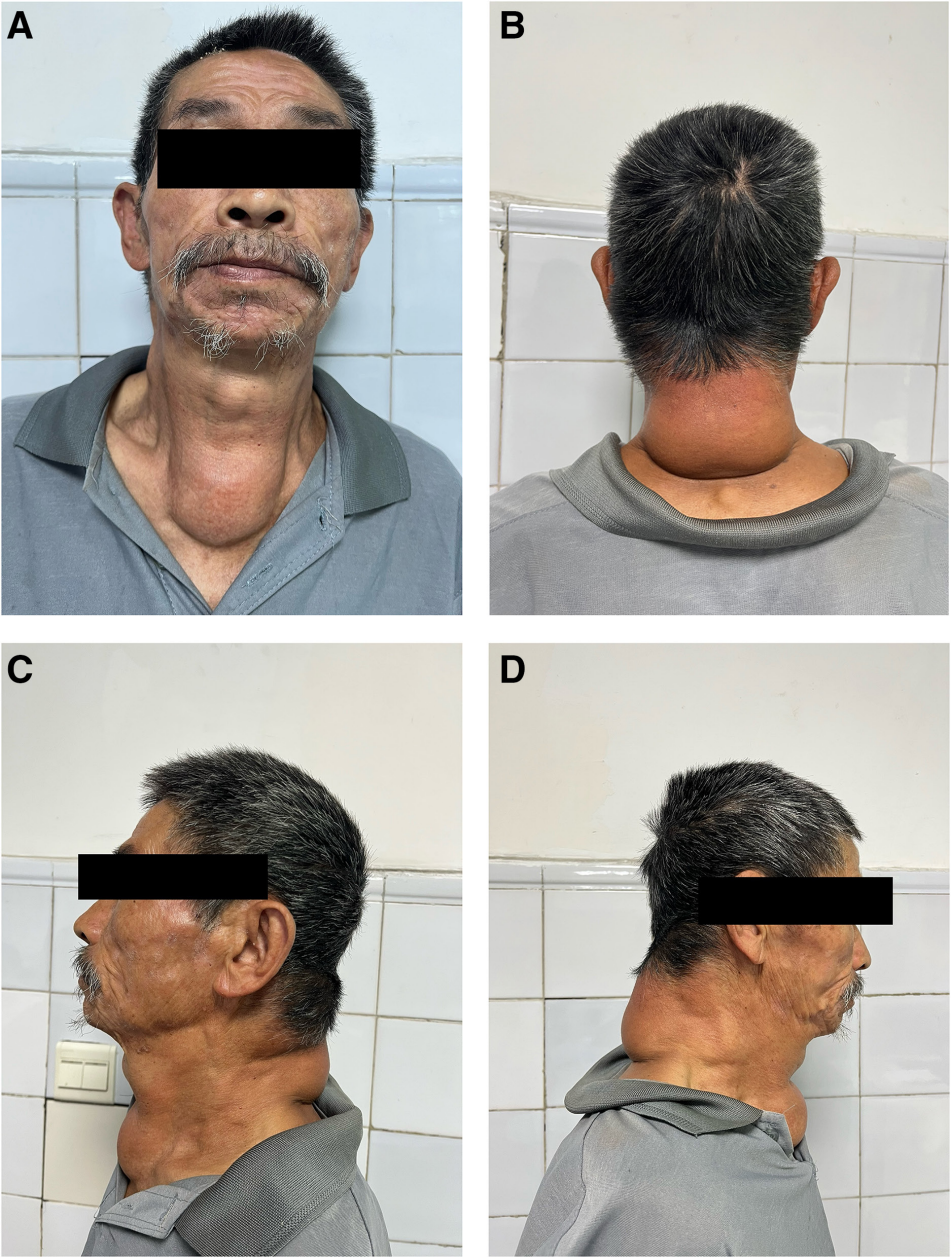
Clinical photographs of the patient showing symmetrical soft tissue swelling in the neck. (A) Anterior view; (B) posterior view; (C) right lateral view; (D) left lateral view. Bilateral, non-tender, diffuse masses in the neck are visible, consistent with the typical presentation of Madelung disease.

The patient was alert and cooperative. Vital signs were stable: temperature 36.5°C; pulse 91 beats/min; respiratory rate 20 breaths/min; blood pressure 145/94 mm Hg. Examination of the neck revealed a soft, non-tender, and well-demarcated anterior mass measuring approximately 5 cm in diameter, accompanied by a larger posterior mass of similar consistency measuring approximately 10 cm, without overlying skin changes (see Fig. 1).

**Figure 2.**
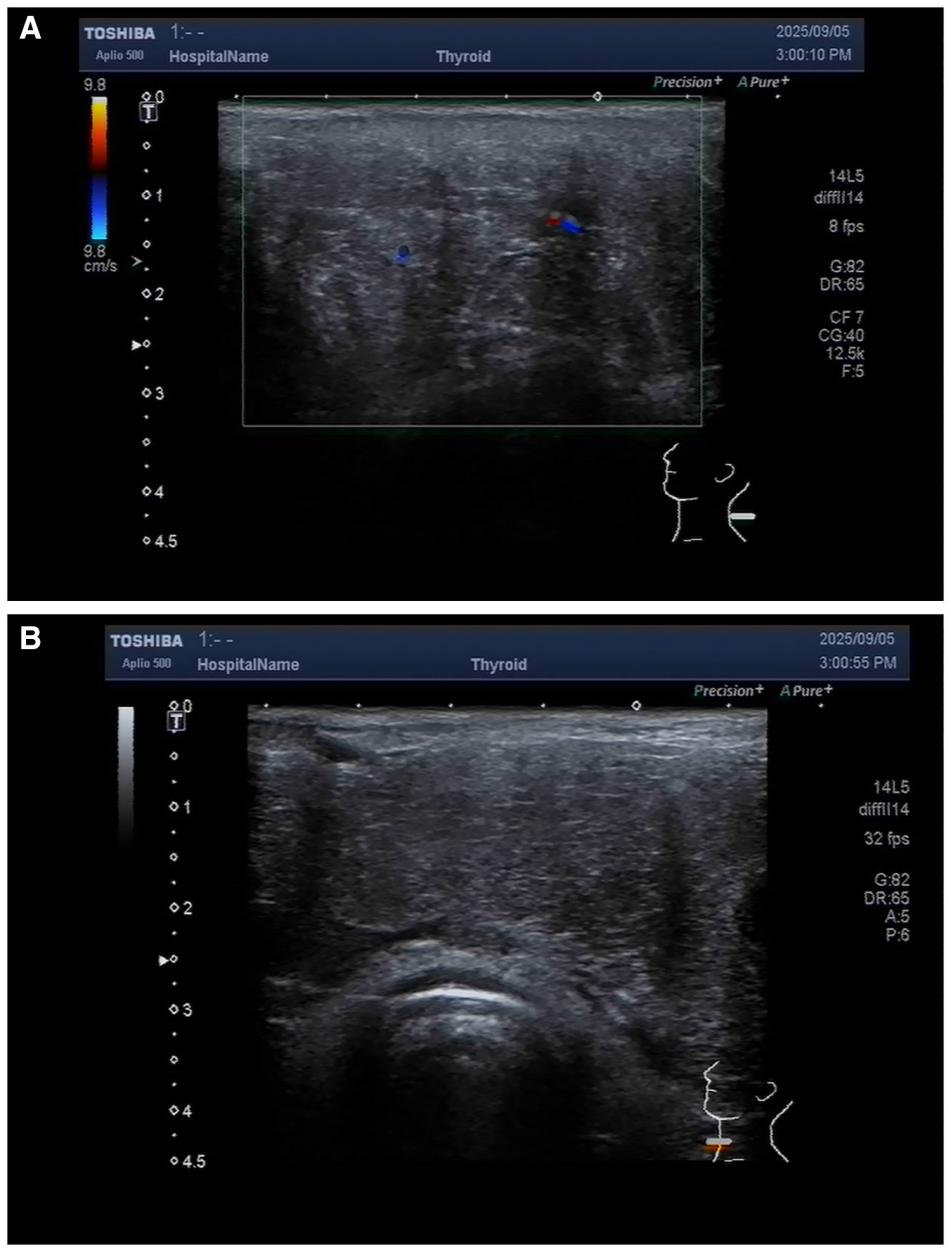
Neck ultrasound images. (A) and (B) demonstrate multiple hyperechoic soft tissue masses in the subcutaneous layer of the neck, without signs of malignant transformation.

**Figure 3.**
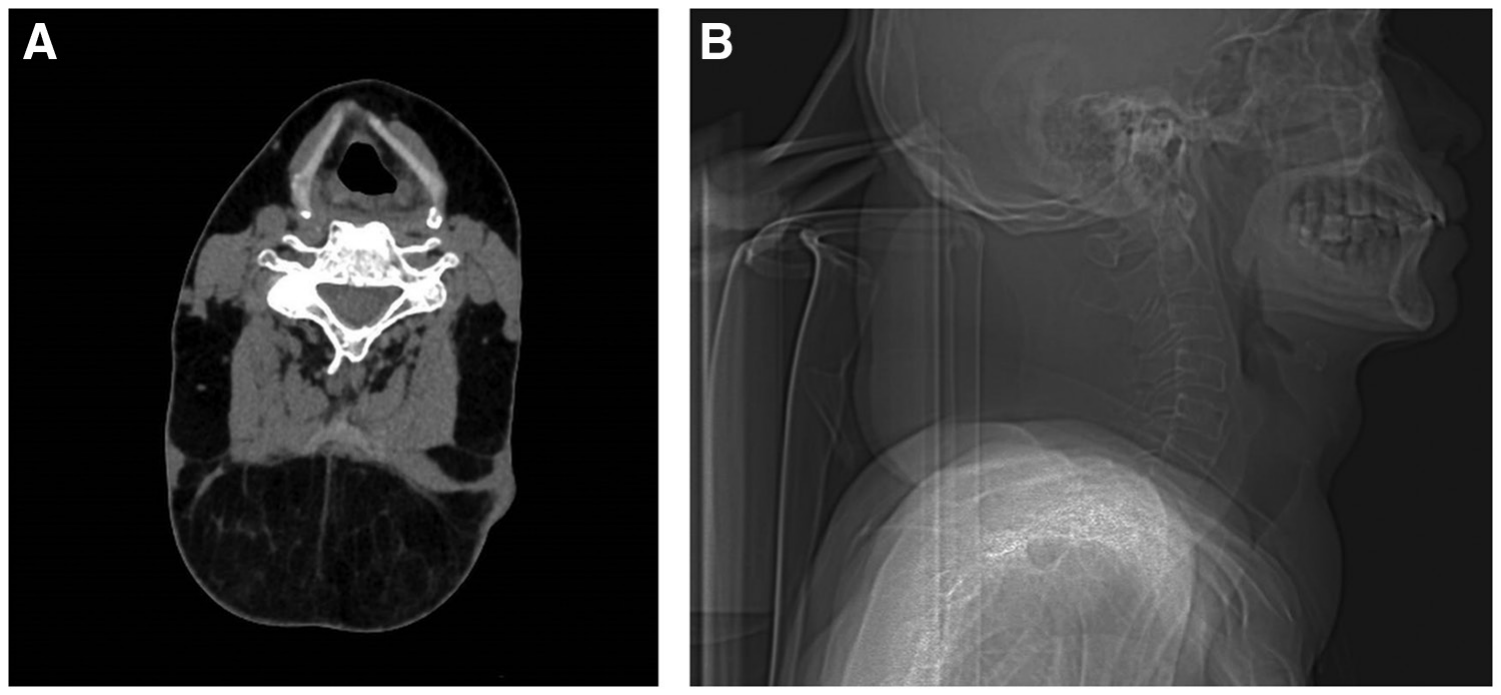
Imaging studies of the neck. (A) Axial computed tomography (CT) scan showing symmetrical, non-encapsulated adipose tissue proliferation in the neck; (B) plain radiograph of the neck showing soft tissue thickening without bony abnormalities.

Laboratory tests revealed a pattern of chronic metabolic disorders, with liver function tests showing markedly elevated aspartate aminotransferase (207 U/L), moderately elevated alanine aminotransferase (42 U/L), and gamma-glutamyl transferase (107 U/L) levels, accompanied by hyperuricemia (uric acid 454 μmol/L). Glucose control was stable (glycated hemoglobin A1c 5.8% [40 mmol/mol]), whereas complete blood count, coagulation function, lipid profile, cardiac markers, and electrolytes were unremarkable. Serum ammonia was within normal reference range, effectively ruling out hepatic encephalopathy. Imaging revealed multiple well-defined avascular hyperechoic masses within the subcutaneous fat layer of the neck. The largest mass, located in the retronuchal region, measured 125 × 27 mm and was consistent with benign lipomatosis (Fig. 2). Abdominal ultrasonography demonstrated hepatic steatosis, cholesterol deposits in the gallbladder, and uric acid crystal deposits in the left kidney. Computed tomography further delineated the extent and symmetry of neck fat deposits (Fig. 3).

The patient’s neurological symptoms promptly resolved after intravenous administration of 50% glucose in the emergency department without any administration of drugs targeted for hepatic encephalopathy such as lactulose or branched-chain amino acids. During hospitalization, management strategies included continuous blood glucose monitoring, hepatoprotective therapy, and lipid-lowering management. Following a detailed discussion regarding cervical lipomatosis, the patient and family opted to defer surgical intervention at this admission. The patient was discharged after achieving stable glycemic control and improved liver function tests.

## 3. Discussion

MD, also known as benign symmetric lipomatosis, is a rare lipid metabolic disorder characterized by the symmetric, non-encapsulated accumulation of abnormal adipose tissue. Epidemiological data indicate that the incidence of this disease is approximately 1 per 25,000 individuals, with a distinct ethnic predilection for populations of European or Mediterranean ancestry. Clinically, MD predominantly affects middle-aged Caucasian males, typically within the age range of 30 to 60 years, and exhibits a striking male predominance with a male-to-female ratio varying from 15:1 to 30:1.^[3]^ Patients with MD frequently manifest multiple systemic complications involving various organ systems, including the endocrine, digestive, circulatory, urinary, and nervous systems, among which endocrine complications are the most prevalent. Notably, MD is relatively uncommon in the Asian population. Owing to the overlapping clinical manifestations with other systemic disorders, MD is prone to misdiagnosis in this region. To date, only a limited number of MD cases have been reported in Asian and African American populations.^[4]^ Based on the primary sites of fat deposition, the disease can be classified into distinct subtypes. The classification system proposed by Schiltz et al in 2018^[5]^ includes: Type Ia, where fat primarily accumulates in the neck; Type Ib, involving the neck, shoulders, and upper arms; Type Ic, extending further to the chest, abdomen, and back beyond Type Ib; Type II, concentrated mainly on the buttocks and thighs; and Type III, presenting as generalized fat accumulation, although the head, forearms, and calves are typically unaffected.

However, the pathogenesis of this condition remains unclear. The literature suggests that MD may be associated with chronic alcohol consumption, endocrine disorders, liver disease, upper respiratory tract malignancies, chromosomal inheritance,^[6]^ metabolic syndromes such as hyperuricemia and hyperlipidemia, type 2 diabetes mellitus, and hypothyroidism.^[7]^ It is currently widely recognized as being closely associated with prolonged, heavy alcohol consumption, with a significantly higher incidence in males than females.^[4,8]^ Furthermore, some studies suggest that its pathogenesis may be linked to certain genetic mutations, such as mitochondrial DNA mutations,^[9]^ Casitas B-lineage lymphoma proto-oncogene B mutations,^[10]^ and lipase E gene mutations.^[11]^ Some studies suggest that pathogenesis may be associated with mitochondrial dysfunction in adipose tissue, reduced cytochrome c oxidase activity, catecholamine-induced fat deposition, and decreased inducible nitric oxide synthase activity.^[12]^ Some scholars report observations of mitochondrial disease in certain patients, including reduced cytochrome c oxidase activity, functional defects in respiratory chain complex IV, irregular red fibers, and multiple deletions of mitochondrial DNA, with rare instances of single large-scale mitochondrial DNA deletions.^[13,14]^

The clinical presentation of MD involves multiple painless masses throughout the body with diffuse symmetrical deposition of subcutaneous adipose tissue. Owing to the complex anatomical structures of the maxillofacial region and neck, the face and neck are the most severely affected areas, exhibiting a high recurrence rate. The characteristic appearance of most MD cases, typically featuring “hamster cheeks,” “horse collars,” and “buffalo humps,” aids diagnosis. As the neck mass increases, patients may progressively develop restricted head rotation, throat compression, narrowing, and dysphagia. This condition is not painful, and the clinical course is slow in most (although not all) cases, with malignant transformation being rare.^[15]^

The diagnosis of MD relies primarily on physical examination and clinical characteristics, supplemented by imaging studies, which correlate with pathological findings.^[8,16]^ Differential diagnosis requires exclusion of other conditions involving excessive adipose tissue, such as morbid obesity, encapsulated lipoma, liposarcoma, Cushing syndrome, salivary gland disorders, goiter, thyroid carcinoma, and cervical cysts.^[17]^

In terms of treatment, as there is no definitive etiologically targeted therapy, all interventions are palliative in nature. The therapeutic objectives are functional restoration and aesthetic improvement, with surgical excision or liposuction currently representing the primary intervention. However, the postoperative recurrence rates remain high.^[18]^ Lipectomy is performed in most cases. Compared with liposuction, it offers a more thorough removal of iatrogenic lesions in adjacent structures and superior control. Liposuction achieves favorable cosmetic outcomes and is simpler and less invasive than lipectomy, although clinical experience remains limited.^[19]^ Conservative management focuses on oral medications to promote lipolysis and improve mitochondrial function, thereby delaying disease progression.^[20]^ Concurrently, reports indicate that intra-adipose therapy may serve as a noninvasive treatment for MD, wherein phospholipid/deoxycholate injections aim to restrict lipoma growth. Scevola et al demonstrated the sustained efficacy of this approach through long-term follow-up studies.^[21]^

The patient was a male with a history of smoking and chronic heavy alcohol consumption, aligning with typical MD demographics. Such characteristics have been documented in prior studies by Quanzhe Liu^[17]^ and Runze Li^[22]^; however, few published cases describe severe hypoglycemic coma occurring in MD patients. Chronic excessive alcohol intake is the primary pathogenic factor for acute alcoholic hypoglycemia in this patient, whereas preexisting hepatic damage from Madelung-related diffuse fatty infiltration serves as a critical predisposing factor aggravating hepatic metabolic insufficiency. Most studies have indicated that chronic alcoholism directly impairs hepatic glycogen storage and gluconeogenic enzyme activity to trigger hypoglycemia.^[23]^ Alcohol also reduces the number and activity of β-adrenergic receptors while promoting triglyceride synthesis. This fat accumulation becomes more pronounced when combined with high-fat dietary intake. Alcohol upregulates lipin-1 gene expression by inhibiting adenosine monophosphate-activated protein kinase and activating sterol regulatory element-binding protein 1. In an alternative pathway, miRNA-217 promotes ethanol-induced fat accumulation by downregulating sirtuin 1.^[24]^ It is noteworthy that alcoholic hypoglycemia in this patient serves as a bridge between MD and hypoglycemic coma. Chronic alcohol consumption causes damage to the hepatic mitochondrial structure and function, leading to a persistent decline in hepatic glycogen synthesis and storage capacity. This reduces the basal activity of enzymes involved in gluconeogenesis, severely impairing the liver’s intrinsic buffering capacity to maintain blood glucose stability. Consequently, both patients with and without chronic liver disease developed hypoglycemic coma following fasting alcohol consumption,^[25]^ and further medical history inquiry confirmed that the coma indeed followed substantial alcohol consumption on an empty stomach. Given the patient’s prior history of alcoholic hypoglycemic coma, clinicians, particularly emergency department physicians, should consider hypoglycemic coma when encountering comatose patients presenting with MD. It is noteworthy that among heavy drinkers, only a minority develop MD, and an even smaller proportion experience extreme metabolic complications such as hypoglycemic coma. This suggests that beyond the direct effects of alcohol, individual genetic susceptibility may be a significant contributing factor. As previously mentioned, the implicated gene mutations predominantly involve pathways that regulate cellular energy metabolism, adipocyte differentiation, and lipolysis. Therefore, it is hypothesized that in addition to prolonged alcohol exposure, the patient in this case may harbor certain as-yet-unidentified genetic variations. This could predispose them to greater mitochondrial dysfunction and increased susceptibility to imbalances in the hepatic energy metabolism pathways. Conducting systematic genetic sequencing and glycometabolic function studies among patients with MD could help elucidate the genetic risk factors for severe hypoglycemic complications, enabling early risk stratification and intervention.

## 4. Clinical recommendations

(1) Lifelong alcohol abstinence is essential to prevent recurrent hypoglycemic episodes. Patients should also avoid prolonged fasting.
(2) A well-balanced diet with sufficient carbohydrate intake is recommended to sustain normal hepatic glycogen reserves.
(3) For first-degree relatives of patients diagnosed with MD, regular physical examination, liver function and blood lipid tests are suggested for early screening of asymptomatic cases.

## Author contributions

**Conceptualization:** Yu Zeng, Song Huang.

**Data curation:** Yu Zeng, Song Huang.

**Formal analysis:** Yu Zeng, Song Huang.

**Investigation:** Yu Zeng.

**Supervision:** Jingyu Hu.

**Visualization:** Jingyu Hu.

**Writing – original draft:** Yu Zeng, Song Huang.

**Writing – review & editing:** Jingyu Hu
